# Insights into the Silver Camphorimine Complexes Interactions with DNA Based on Cyclic Voltammetry and Docking Studies

**DOI:** 10.3390/molecules30132817

**Published:** 2025-06-30

**Authors:** Joana P. Costa, Gonçalo C. Justino, Fernanda Marques, M. Fernanda N. N. Carvalho

**Affiliations:** 1 Centro de Química Estrutural, Institute of Molecular Sciences, Instituto Superior Técnico, Universidade de Lisboa, Av. Rovisco Pais, 1049-001 Lisboa, Portugalgoncalo.justino@tecnico.ulisboa.pt (G.C.J.); 2 Escola Superior de Tecnologia do Barreiro, Instituto Politécnico de Setúbal, R. Américo da Silva Marinho, 2839-001 Lavradio, Portugal; 3 Centro de Ciências e Tecnologias Nucleares, Departamento de Engenharia e Ciências Nucleares, Instituto Superior Técnico, Universidade de Lisboa, Estrada Nacional 10 (Km 139,7), Bobadela, 2695-066 Loures, Portugal; fmarujo@ctn.tecnico.ulisboa.pt

**Keywords:** cyclic voltammetry, silver camphorimine complexes, DNA, docking studies, cytotoxicity

## Abstract

Cyclic voltammetry (CV) is an accessible, readily available, non-expensive technique that can be used to search for the interaction of compounds with DNA and detect the strongest DNA-binding from a set of compounds, therefore allowing for the optimization of the number of cytotoxicity assays. Focusing on this electrochemical approach, the study of twenty-seven camphorimine silver complexes of six different families was performed aiming at detecting interactions with calf thymus DNA (CT-DNA). All of the complexes display at least two cathodic waves attributed respectively to the Ag(I)→Ag(0) (higher potential) and ligand based (lower potential) reductions. In the presence of CT-DNA, a negative shift in the potential of the Ag(I)→Ag(0) reduction was observed in all cases. Additional changes in the potential of the waves, attributed to the ligand-based reduction, were also observed. The formation of a light grey product adherent to the Pt electrode in the case of {Ag(OH)} and {Ag_2_(µ-O)} complexes further corroborates the interaction of the complexes with CT-DNA detected by CV. The morphologic analysis of the light grey material was made by scanning electronic microscopy (SEM). The magnitude of the shift in the potential of the Ag(I)→Ag(0) reduction in the presence of CT-DNA differs among the families of the complexes. The complexes based on {Ag(NO_3_)} exhibit higher potential shifts than those based on {Ag(OH)}, while the characteristics of the ligand (^A^L-Y, ^B^L-Y, ^C^L-Z) and the imine substituents (Y,Z) fine-tune the potential shifts. The energy values calculated by docking corroborate the tendency in the magnitude of the interaction between the complexes and CT-DNA established by the reaction coefficient ratios (Q_[Ag-DNA]_/Q_[Ag]_). The molecular docking study extended the information regarding the type of interaction beyond the usual intercalation, groove binding, or electrostatic modes that are typically reported, allowing a finer understanding of the non-covalent interactions involved. The rationalization of the CV and cytotoxicity data for the Ag(I) camphorimine complexes support a direct relationship between the shifts in the potential and the cytotoxic activities of the complexes, aiding the decision on whether the cytotoxicity of a complex from a family is worthy of evaluation.

## 1. Introduction

Cancer belongs to a group of diseases often originated from DNA mutations that lead to genomic instability, resulting in an increase in the amount of abnormal cells due to DNA damage [1]. The screening for new compounds as effective anticancer drugs is a long process with several crucial steps, which include the analysis of the cytotoxic properties and identification of cancer cell targets. DNA is often a primary cellular target for anticancer drugs. Analysis of the cytotoxic properties through determination of the half-maximal inhibitory concentrations (IC_50_) is one of the initial steps that require labs, skills and time and which incur a considerable cost.

Metal-based drugs belong to a potential class of anticancer drugs acting by specific pathways. Due to their different physiochemical and biological properties, metal drugs bind to different cellular targets and therefore present specific mechanism of action [2]. However, this is a long process that starts with the synthesis and chemical/structural characterization of the compounds and continues with the in vitro evaluation of the cytotoxicity and elucidation of the mechanisms of action, before a lead compound fulfils the requirements to proceed to in vivo studies. Therefore, shortening some of these steps is highly desirable for focused scientific results and in terms of costs. Among coordination compounds, cisplatin and its analogous platin coordination compounds, display a high rate of success in cancer treatment, underscoring the effectiveness of such compounds as anticancer agents and driving the search for other coordination compounds that may reduce side effects and/or acquired resistance [3,4,5,6].

The study of the interaction of metal complexes with nucleic acids (e.g., DNA), in order to predict specific site-selective interactions and possible binding modes, involves identification of DNA as a drug target from the many possible targets in the cell. To do so, techniques such as UV–vis spectrometry, fluorescence and circular dichroism spectroscopy have been used, as well as more advanced methods such as electrophoresis, atomic force microscopy, electrochemistry and complementary in silico methods [7]. These techniques cannot, per se, clearly interpret the mode of complex–DNA interaction. However, they can combine and help to clarify the drug target and mechanisms of action. In this context, electrochemical methods can be used to identify specific interactions of compounds with DNA and get insights into the redox mechanisms of cell homeostasis [8,9]. Coordination compounds combine typical interactions of organic molecules with DNA with those of metals which often involve electron transfer processes. Cyclic voltammetry (CV) is an accessible and quite versatile electrochemical technique by which to signalize such interactions through changes in the redox potentials (ΔE) and/or current intensity (ΔI) [10,11,12], thus becoming a fast-track technique to comprehend DNA electron transfer processes and get insights into the type of mechanism and thermodynamic parameters (e.g., K) involved in compound–DNA interactions [13,14,15,16]. The recognition of a new compound as a certified drug for cancer treatment is a process with many steps, one of which includes the identification of the mechanism of cell death. Molecules and organic, inorganic or coordination compounds can interact with DNA through covalent bonding and electrostatic interactions such as intercalation or groove binding [17,18].

We have previously addressed the search for new, potentially biologically active, compounds by synthesizing camphorimine silver, gold and copper complexes and evaluating their cytotoxic activity against several types of cancer cells [19]. A step forward is now given towards the elucidation of the type of interaction established between a selection of silver camphorimine complexes and DNA. The study further aims at contributing to the early in vitro stages of drug development by using cyclic voltammetry as an accessible screening tool of drug–DNA interaction.

## 2. Results

The silver camphorimine compounds whose redox properties are discussed below were assessed towards osteosarcoma (HOS) or ovarian cancer (A2780 and OVCAR3) cell lines, using the MTT (3-(4,5-dimethylthiazol-2-yl)-2,5-diphenyltetrazolium bromide) method for cytotoxicity assessment [19]. All compounds display anticancer activity, with cytotoxicity varying with the type of camphorimine ligand. An insight into the type of interactions established between the Ag(I) complexes and DNA is now addressed by cyclic voltammetry and complementary docking studies.

### 2.1. Redox Properties {Ag(NO_3_)} Camphor-Derived Complexes

**Figure 1:**
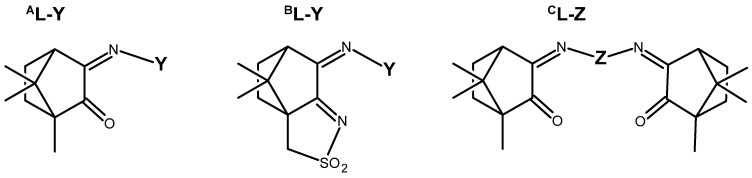
Camphorimine compounds used as ligands for Ag(I) complexes. Y and Z groups are displayed in Table 1, Table 2, Table 3, Table 4, Table 5 and Table 6.

The redox properties of Ag(I) complexes with camphor-derived ligands (^A^L-Y, ^B^L-Y, ^C^L-Z, Figure 1) were studied (or revisited) by cyclic voltammetry (CV) in acetonitrile using a Pt wire electrode. The redox properties of the camphor-derived coordination compounds mostly rely on the characteristics of the metal site {AgX} (X^−^ = NO_3_, OH or µ-O), but the ligands fine-tune the potentials.

Four sets of silver nitrate complexes of general formula [Ag(NO_3_)(^A^L-Y)_2_], [Ag(NO_3_)(^A^L-Y)] (Table 1), [Ag(NO_3_)(^C^L-Z)] (Table 2), and [Ag(NO_3_)(^B^L-Y)_2_] (Table 3); two sets of silver hydroxy complexes [Ag(OH)(^A^L)] (Table 4) and [Ag(OH)(^B^L-Y)] (Table 5); and two sets of complexes of formula [{Ag(^A^L-Y)}_2_(μ-O)] and [{Ag(^B^L-Y)_2_}_2_(μ-O)] (Table 6) were studied. For comparative purposes the redox properties of the complexes’ precursors, silver nitrate, Ag(NO_3_) ($E_{p}^{red}$ = 0.18 V, vs. SCE), and silver acetate, Ag(CH_3_COO) ($E_{p}^{red}$ = 0.088 V, vs. SCE), were studied under the same experimental conditions. The redox data are grouped according to the characteristics of the metal site, i.e., Table 1, Table 2 and Table 3 refer to the nitrate type {Ag(NO_3_)} complexes, Table 4 and Table 5 to the hydroxy type {Ag(OH)} complexes, and Table 6 to the O-bridged {Ag_2_(μ-O)} complexes.

**Table 1:** Cyclic voltammetry data ^a^ for complexes [Ag(NO_3_)(^A^L-Y)_n_] (n = 2, **1**–**8**; n = 1, **9**).

		EpredI	E1/2redII	$E_{p}^{ox}$
Complex	^A^L-Y	Volt		
**1**	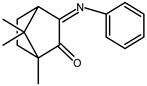	0.12 ^b^	−1.62	1.63
**1** + DNA		−0.046	−1.65 ^c^	—
**2**	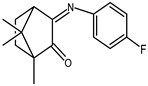	0.14	−1.58	1.7
**2** + DNA		0.065	−1.45	—
**3**	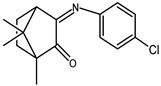	0.12 ^b^	−1.27	1.58
**3** + DNA		0.004	−1.26	—
**4**	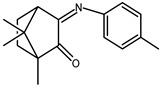	0.10	−1.65	1.6
**4** + DNA		−0.058	−1.50	1.61
**5**	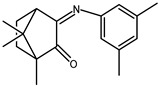	0.11 ^b^	−1.60	1.49
**5** + DNA		−0.063	−1.54	1.62
**6**	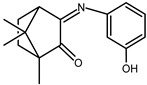	0.074 ^b^	−1.64	0.95
**6** + DNA		−0.076	−1.67 ^c^	1.08
**7**	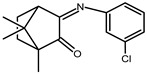	0.014	−1.42 ^c^	1.19
**7** + DNA		−0.16	−1.29 ^c^	1.17
**8**	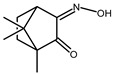	0.10 ^b^	−1.65	1.6
**8** + DNA		0.040	−1.85 ^c^	—
**9**	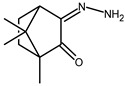	0.12 ^d^	—	1.75
**9** + DNA		0.041	—	—

^a^ Values in volt ± 10 mV, vs. sce measured at 200 mV/s in Bu_4_NBF_4_/CH_3_CN using as internal reference [Fe(η^5^-C_5_H_5_)_2_]^0/+^ (0.382 volt). ^b^ Ref. [20]. ^c^ Irreversible wave ($E_{p}^{red}$). ^d^ Ref. [21].

**Table 2:** Cyclic voltammetry data ^a^ obtained for bicamphor complexes [Ag(NO_3_)(^C^L-Z)].

		EpredI	E1/2redII	$E_{p}^{ox}$
	^C^L-Z	Volt		
**10**	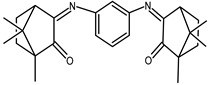	0.14	−1.43	1.63
**10** + DNA		−0.030	−1.38	—
**11**	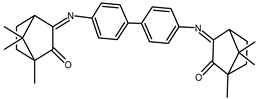	0.11	−1.55	1.33
**11**+ DNA		−0.01	−1.49	1.42

^a^ Values in volt ± 10 mV, vs. sce measured at 200 mV/s in Bu_4_NBF_4_/CH_3_CN using [Fe(η^5^-C_5_H_5_)_2_]^0/+^ (0.382 volt) as internal reference.

**Table 3:** Cyclic voltammetry data ^a^ for camphor sulfonimine [Ag(NO_3_)(^B^L-Y)_2_] (**12**–**17**) complexes and AgNO_3_.

		EpredI	EpredII	$E_{p}^{ox}$
COMPLEX	^B^L-Y	Volt		
**12**	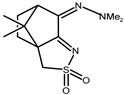	0.17	−1.54	1.34
**12** + DNA		0.11	−1.36	—
**13**	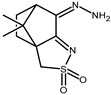	0.13	−1.39	1.67
**13** + DNA		0.036	−1.55	1.54
**14**	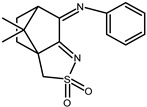	0.13	−1.13 −1.52	1.89
**14** + DNA		0.010	−1.14	—
**15**	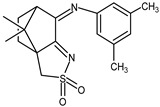	0.14	−1.18	—
**15** + DNA		0.04	−1.06	—
**16**	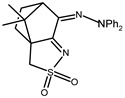	0.15	−1.35 −1.85	1.28
**16** + DNA		0.090	−1.25	—
**17**	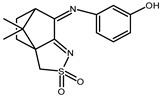	0.080	−1.13 −1.23	1.26 1.57
**17** + DNA		−0.040	−0.93 −1.25	—
AgNO_3_	—	0.18	—	2.01
AgNO_3_ + DNA	—	0.041		

^a^ Values in volt ± 10 mV, vs. sce measured at 200 mV/s in Bu_4_NBF_4_/CH_3_CN using [Fe(η^5^-C_5_H_5_)_2_]^0/+^ (0.382 volt) as internal reference.

**Figure 2:**
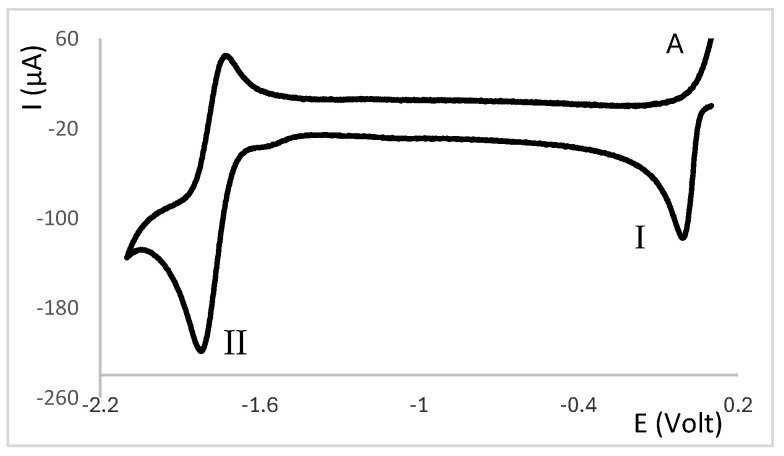
Partial cyclic voltammogram of [Ag(NO_3_)(^A^L-Y)_2_] (**4**, ^A^L-Y; Y = 4-CH_3_C_6_H_4_) showing cathodic (I and II) and adsorption (A) waves.

All complexes based on the central core {AgNO_3_}, independent of the camphor-derived ligands (^A^L-Y, Table 1; ^C^L-Z, Table 2; ^B^L-Y, Table 3), display one irreversible cathodic wave at potentials from zero to 0.17 volts (wave I, Figure 2), which is attributed to Ag(I)→Ag(0) reduction. Upon wave I, on the reverse scan, the adsorption wave (A, Figure 2) confirms the formation of silver metal at the electrode double layer. In all cases, the potential of wave I is lower than that of the precursor silver nitrate (0.18 V). Complexes **6**, **7** and **17**, with ligands (^A^L-Y, ^B^L-Y), which in turn have aromatic groups (Y) with substituents at the *meta* position, display the lowest potentials.

**Figure 3:**
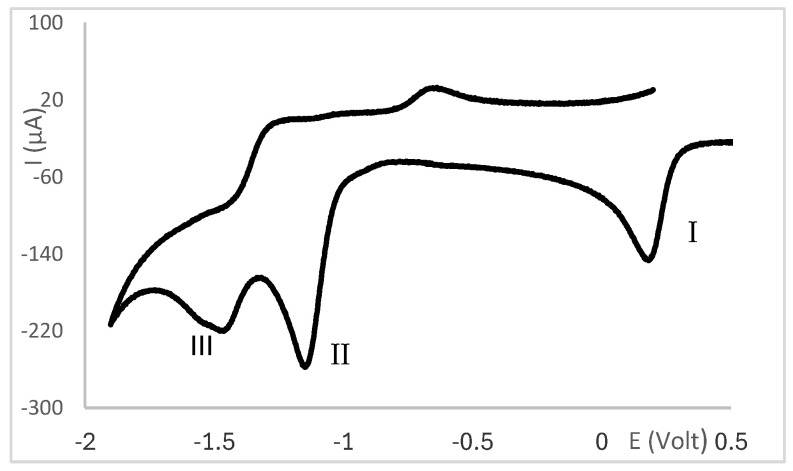
Cyclic voltammogram of [Ag(NO_3_)(^B^L-Y)_2_] (**14**, ^B^L-Y; Y = C_6_H_5_) obtained at 200 mV/s in Bu_4_NBF_4_/CH_3_CN using a Pt wire working electrode.

A second cathodic wave of ca. double the intensity of wave I is observed (wave II, Figure 2) at potentials lower than those of wave I. Wave II is attributed to ligand-based reduction process. The cyclic voltammograms of complexes **14**, **16** and **17** with ^B^L-Y ligands display additional cathodic waves attributed to ligand-based processes (III, Figure 3). Almost all complexes display oxidation waves attributed to the ligands, but oxidation processes are not addressed in this study.

### 2.2. Redox Properties of {Ag(OH)} and {Ag_2_(µ-O)} Camphor-Derived Complexes

From the reaction of silver acetate with ^A^L-Y and ^B^L-Y (Figure 1) camphor derivatives, the following four types of compounds were obtained: [Ag(OH)(L-Y)] (L = ^A^L-Y or ^B^L-Y); [{Ag(^A^L-Y)}_2_(µ-O)] and [{Ag(^B^L-Y)_2_}_2_(µ-O)] [21].

All of the complexes, either hydroxide **18**–**23** (Table 4 and Table 5) or oxide **24**–**27** (Table 6), display irreversible cathodic waves at potentials close to zero volts within values that are lower than those measured for the {Ag(NO_3_)} complexes, in line with the potentials of the corresponding precursors (Table 3 and Table 6).

**Table 4:** Cyclic voltammetry data ^a^ for camphorimine [Ag(OH)(^A^L-Y)] (**18**–**20**) and [Ag(OH)(^C^L-Z)] (**21**) complexes.

COMPLEX	^A^L-Y	EpredI	EpredII	$E_{p}^{ox}$
		(Volt)		
**18**	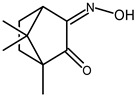	0.053	—	0.41
**18** + DNA		−0.001	—	—
**19**	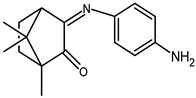	0.034	−1.63	0.84
**19** + DNA		0.020	−1.51	—
**20**	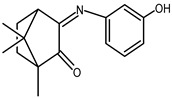	0.062	−1.62	1.57
**20** + DNA		0.036	−1.42	—
	^C^L-Z			
**21**	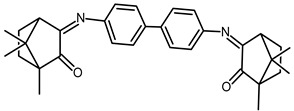	−0.014	−1.54	1.40
**21** + DNA		−0.044	−1.46	1.15

^a^ Values in volt (±10 mV) vs sce, measured at 200 mV/s.

**Table 5:** Cyclic voltammetry data ^a^ for camphor sulfonimine [Ag(OH)(^B^L-Y)] (**22**–**23**) complexes.

		EpredI	EpredII	$E_{p}^{ox}$
COMPLEX	^B^L-Y	Volt		
**22**	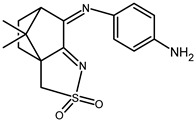	0.090 ^b^	−1.33 −1.85 ^c^	0.82 1.69
**22** + DNA		0.062	−1.61	0.69
**23**	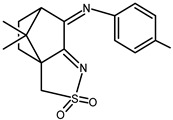	0.11 ^b^	−1.19 −1.66 ^c^	1.69
**23** + DNA		0.074	−1.10	—

^a^ Values in volt (±10 mV) vs sce, measured at 200 mV/s. ^b^ Observed upon anodic scan. ^c^ Some reversibility, low intensity.

**Table 6:** Cyclic voltammetry data ^a^ for camphorimine [{Ag(^A^L-Y)}_2_(µ-O)] (**24**–**25**), camphor sulfonimine [{Ag(^B^L-Y)_2_}_2_(µ-O)] (**26**) and bicamphor [{Ag_2_(^C^L-Z)} (µ-O)] (**27**) complexes.

		EpredI	EpredII	$E_{p}^{ox}$
COMPLEX	^A^L-Y		Volt	
**24**	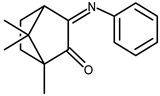	−0.021	−1.63	1.70
**24 +** DNA		−0.11	−1.36	—
**25**	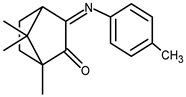	0.024	−1.54	—
**25 +** DNA		−0.014	−1.30	—
	^B^L-Y			
**26**	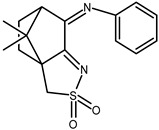	0.010 V	−1.20	—
**26 +** DNA		−0.004	−1.17	—
	^C^L-Z			
**27**	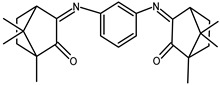	−0.056	−1.59	1.03
**27 +** DNA		−0.106	−1.33	1.2
Ag(CH_3_COO)		0.088		1.16
Ag(CH_3_COO) + DNA		0.084		1.13

^a^ Values in volt (±10 mV) vs sce, measured at 200 mV/s.

As an overall, the potential of the Ag(I)→Ag(0) reduction process falls within a narrow potential range either in the case of the nitrate or the hydroxide or oxide complexes, independently of the camphor scaffold type (^A^L, ^B^L, or ^C^L) and of the imine substituent Y. These results show that the camphor backbone and Y substituent play only a minor role in setting the redox potential. However, the nature of the ancillary anion at the silver site drives the electrochemical behavior used to probe DNA binding.

### 2.3. Electrochemical Behaviour of Ag(I) Camphorimine Complexes in the Presence of CT-DNA Solution

The purpose for the systematic study of the electrochemical properties of these 27 Ag(I) camphorimine complexes was to create a sufficiently large pool of redox data to support cyclic voltammetry as an accessible method to identify interactions of the complexes with DNA. Such interactions can be detected by changes in the intensity of the redox waves and/or shifts in their peak potentials. According to Bard et al. positive shifts in the potential of the waves indicate that the complexes intercalate into the DNA double-strand, while negative shifts indicate groove-binding interactions [17].

**Figure 4:**
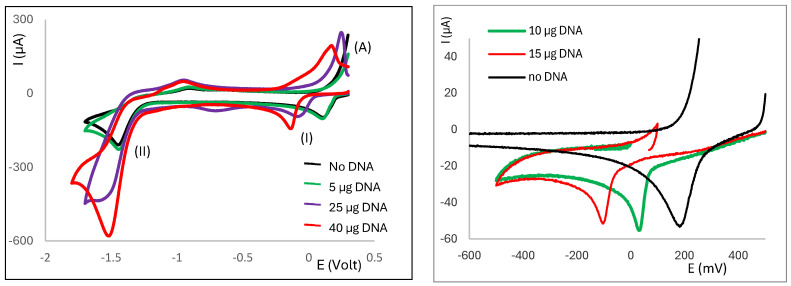
Partial cyclic voltammograms of [Ag(NO_3_)(^B^L-Y)_2_] (Y = NH_2_) (**left**) and [Ag(NO_3_)(^A^L-Y)_2_] (Y = NH_2_) (**right**) in the absence (black) or presence of CT-DNA (variable amounts).

The Ag(I) camphor-derived complexes are not soluble in water, while the deoxyribonucleic acid sodium salt from calf thymus (CT-DNA) is not soluble in acetonitrile, which is the solvent commonly used to study the redox properties of these complexes. Thus, a stock solution of CT-DNA (500 μg/1.00 mL) in borate buffer (pH 7.5) was prepared and variable quantities of that solution were added to the solution of the complexes in the electrochemical cell. Typically, the process begins with the addition of 10 μL (5 μg) of the CT-DNA solution to the cell solution (2.5 mL) of the complex (1–2 mM). In most cases, no effective changes in the cyclic voltammograms were observed when adding the initial (10 μL) amount of CT-DNA solution, in agreement with the lack of sensible changes being introduced in the redox properties of the complexes. However, increasing the amount of CT-DNA in the cell solution is responsible for a shift in the potential of wave I (attributed to the Ag(I)→Ag(0) reduction) to lower values. As representative examples, the partial cyclic voltammograms of [Ag(NO_3_)(^B^L-Y)_2_] (Y = NH_2_,) (Figure 4 left) and [Ag(NO_3_)(^A^L)_2_] (Y = NH_2_) (Figure 4 right) are displayed in the absence (black line) and presence of variable amounts of CT-DNA.

**Figure 5:**
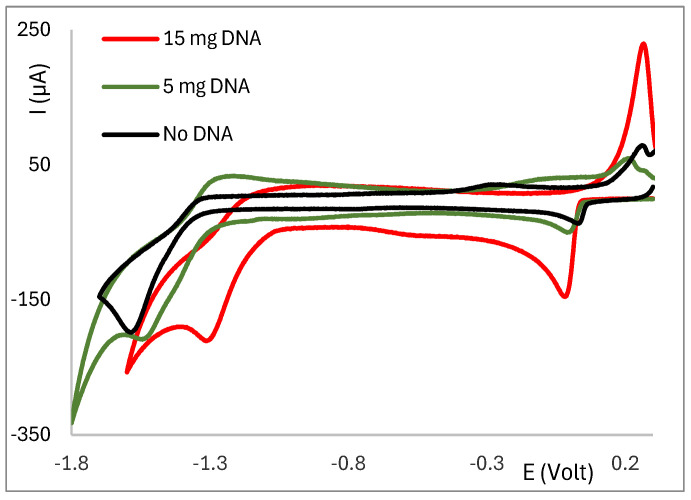
Cyclic voltammogram of [{Ag(^A^L)}_2_(µ-O)] (Y = 4-CH_3_C_6_H_5_) obtained at 200 mV/s in Bu_4_NBF_4_/CH_3_CN in the absence (black) or presence of CT-DNA (variable amounts).

The behavior of wave II (attributed to a ligand-based process) shows two distinct trends: (i) the potential changes only slightly, whereas the intensity of the wave increases (Figure 4 left), or (ii) the potential shifts to more positive values—an effect more pronounced in complexes based on {Ag(OH)} and {Ag_2_(µ-O)} for which the intensity of the wave remains nearly constant. In the second case, the intensity of wave I concomitantly increases (Figure 5). Complexes **13**, **14**, and **22**, which display two ligand-based cathodic processes, do not fit any of the patterns.

The increase in the intensity of wave I was unexpected in view of most published electrochemical data on the interaction of compounds with DNA [16,22,23]. Typically, a decrease is reported which is attributed to the lower diffusivity of the compound–DNA entity compared with the free compound [17]. However, in Ag(I) camphorimine complexes an increase in the intensity of wave I was observed. Such a pattern has also been reported for the interaction of aripiprazole with DNA [24]. In both studies the working electrode was a Pt wire, in contrast with typically used glassy carbon electrode, suggesting that the characteristics of the electrode play a relevant role in the outcome of the studies.

## 3. Discussion

The electrochemical behavior of the six families of the complexes under study shows that, in the presence of CT-DNA, the potential of the Ag(I)→Ag(0) process (wave I) shifts to more negative values in all cases. Such a shift is considerably more significative in the case of the silver nitrate {Ag(NO_3_)} (**1**–**17**) complexes than in the case of {Ag(OH)} (**18**–**23**) or {Ag(µ-O)} (**24**–**26**) complexes (Table 7). The [Ag(NO_3_)(^A^L-Y)] (**6**, **7**) complexes with *meta* substituents (Y) at the aromatic imine group of the ligand (^A^L-Y) display the biggest differences in the potential of wave I (Table 7), pointing to a significant effect of the stereochemical characteristics of the substituent (Y) in the interaction of the complexes with DNA.

While displaying a small shift in the potential upon addition of CT-DNA, complexes **18**–**26** display an increase in the intensity of wave I (Figure 5). Such a pattern is attributed to the build-up of the product of interaction on the Pt electrode supported by the appearance of light grey filaments.

The ligand-based processes (wave II) display a trend that is opposite to that of wave I. The shift is smaller in the case of complexes based on the {Ag(NO_3_)} core than in {Ag(OH)} or {Ag_2_(µ-O)} (Table 7).

**Table 7:** Shifts in the potential of waves I and II in the presence or absence of CT-DNA and related reaction coefficient ratios.

Complex	Δ(^I^E_DNA_ − ^I^E)	Q_[Ag]+DNA_/Q_[Ag]_	E ^a^	Complex	Δ(^I^E_DNA_ − ^I^E)	Q_[Ag]+DNA_/Q_[Ag]_	E ^a^
	mV		kcal/mol		mV		kcal/mol
**1**	−166	6.4 × 10^2^	−252.1	**15**	−100	4.9 × 10^1^	
**2**	−75	1.9 × 10^1^		**16**	−60	1.0 × 10^1^	−277.6
**3**	−116	9.2 × 10^1^		**17**	−120	1.1 × 10^2^	
**4**	−158	4.7 × 10^2^	−246.5	**18**	−54	8.2	
**5**	−173	8.5 × 10^2^	−274.7	**19**	−14	1.7	−219.8
**6**	−150	3.4 × 10^2^	−280.7	**20**	−26	2.7	
**7**	−174	8.8 × 10^2^	−276.5	**21**	−30	3.2	−238.6
**8**	−60	1.0 × 10^1^		**22**	−28	3.0	
**9**	−79	2.2 × 10^1^		**23**	−36	4.1	−236.9
**10**	−143	2.6 × 10^2^		**24**	−89	3.2	
**11**	−116	1.1 × 10^2^	−252.9	**25**	−38	4.4	
**12**	−60	1.0 × 10^1^	−224.8	**26**	−6	1.3	
**13**	−94	3.9 × 10^1^		**27**	−50	7.0	
**14**	−120	1.1 × 10^2^	−241.2				

^a^ Binding energy as computed by docking.

The magnitude of the shifts in the potential of the Ag(I)→Ag(0) reduction, in the presence of DNA, show that the metal center {AgX} (X = NO_3_^−^, OH^−^, (µ-O)^−^) drives the process and eventually the type of interaction with DNA.

**Figure 6:**
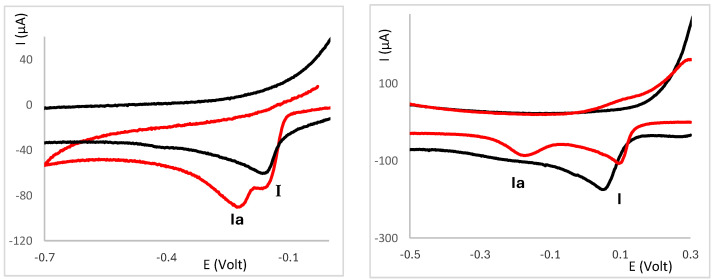
Partial cyclic voltammograms of complexes **18** (**left**) and **23** (**right**) in the absence (black line) and presence of CT-DNA (red line).

As mentioned above, nitrate complexes (**1**–**17**) display considerably larger shifts than the hydroxy (**18**–**23**) or oxide (**24**–**26**) complexes in the potential of wave I. Whether such shifts are due to an outer-sphere type interaction or the formation of a new species was partially clarified by the appearance of a second irreversible cathodic wave (Figure 6, Ia) in the cyclic voltammograms of complexes [Ag(NO_3_)(^A^L-Y)_2_] (Y = Ph, **1**), [Ag(OH)(^A^L-Y)] (Y = OH, **18**) or [Ag(OH)(^B^L-Y)] (Y = 4-CH_3_C_6_H_4_, **23**), consistent with the formation of a new species resulting from the interaction of the complexes with CT-DNA.

### 3.1. Correlation Between Potential and Reaction Coefficient

The potential of the redox processes in the absence (E_[Ag]_) and presence (E_[Ag]+DNA_) of CT-DNA can be related with the equilibrium constant [13] or reaction coefficient (Q) through the Nernst equation (E_[Ag]+DNA_ − E_[Ag]_ = $\frac{RT}{nF}$log_10_(Q_[Ag]_/Q_[Ag]+DNA_)). Because, in the Ag(I)→Ag(0) process, one electron *per* mole is transferred, at 25 °C the equation becomes Q_[Ag]_/Q_[Ag]+DNA_= $10^{\frac{E_{[Ag]+DNA}-E_{\left[Ag\right]}}{0.0591}}$. By application of this relationship, the Q_[Ag]+DNA_/Q_[Ag]_ values were calculated for all complexes (Table 7). Nitrate complexes (**1**–**17**) display Q_[Ag]+DNA_/Q_[Ag]_ values that differ from the hydroxide (**18**–**23**) and oxide complexes (**24**–**27**) by up to two orders of magnitude (Table 7). Another criterium to evaluate thermodynamic parameters would be through the correlation between the decrease in the intensity of the wave and the equilibrium constant (log (1/[DNA]) = log K + log (I/(I_0_ − I)) [18,25]. Such criterium cannot be used in this study because no decrease in the current intensity (I) was observed. On the contrary, the intensity of wave I remained almost constant (**1**–**17**) or even increased (**18**–**27**) due to product build-up on the Pt electrode. This product could be manually removed as a whitish fibber, whose morphology was analyzed by SEM.

The calculated Q_[Ag]+DNA_/Q_[Ag]_ values reveal distinct magnitudes (Table 7) and conceivably different types of interaction of the silver camphorimine complexes with CT-DNA.

Docking studies were carried out to get insights into the strength and the types of interaction of the complexes with CT-DNA.

### 3.2. Docking Studies

The docking studies focused on elucidating the binding modes, interaction strengths, and preferred binding sites on DNA. Aiming at that, a selection was made to cover the types of complexes under study. The binding of the Ag(I) complexes to DNA was explored using Hex 8.0.0 on a 16-mer double-stranded DNA model (5′-GCTGGATTAATCCAGC-3′), which encompasses all possible consecutive base combinations (based on PDB ID 8V5J).

**Figure 7:**
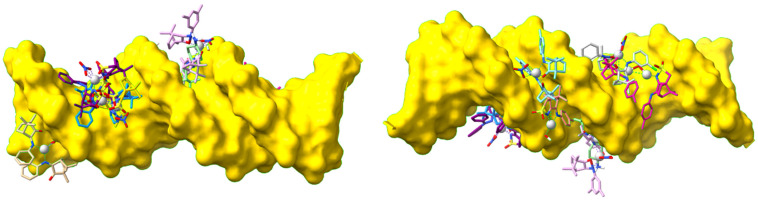
Molecular docking of Ag(I) camphorimine complexes to DNA. The DNA is shown as a gold surface, while the top-ranked docking poses for each Ag(I) complex are represented as sticks, with Ag atoms highlighted as spheres. This visualization illustrates the preferred binding orientations and potential interaction sites of the Ag complexes with the DNA structure.

The camphorimine complexes were found to bind at the surface of the DNA strand (Figure 7), with no evidence of intercalation. Such findings were expected according to the non-planar global geometry of the complexes and are in line with Bard’s predictions [14].

To complement the electrochemical evaluation of DNA binding, molecular docking studies were carried out to assess the preferred binding modes, interaction types, and relative affinities of representative Ag(I) complexes toward a 16-mer double-stranded B-DNA model (PDB ID 8V5J) [26]. A blind, rigid-body docking approach using Hex 8.0.0 [27,28] was employed to explore the full DNA surface, with ranking based on shape and electrostatic complementarity. The resulting docked poses were analyzed to identify key non-covalent interactions, including hydrogen bonding, hydrophobic contacts, and bridging electrostatics. Notably, no intercalation was observed among the top-ranked poses, consistent with the non-planar geometry of the complexes. Docking energies were then compared across ligand families and co-ligands to interpret how steric and electronic features modulate DNA affinity. The results are discussed below in relation to complex geometry, binding site, and the nature of ligand–DNA interactions.

Complexes **1**, **4**, **5**, **19** and **23** bind to the minor groove, while complexes **7**, **25**, **12**, **14** and **16** preferentially bind to the major groove. This distribution broadly reflects the overall geometry of the complexes, as minor groove binders are generally more elongated, and major groove binders tend to be bulkier, restricting their ability to fit into the minor groove but enabling interaction within the wider major groove.

**Figure 8:**
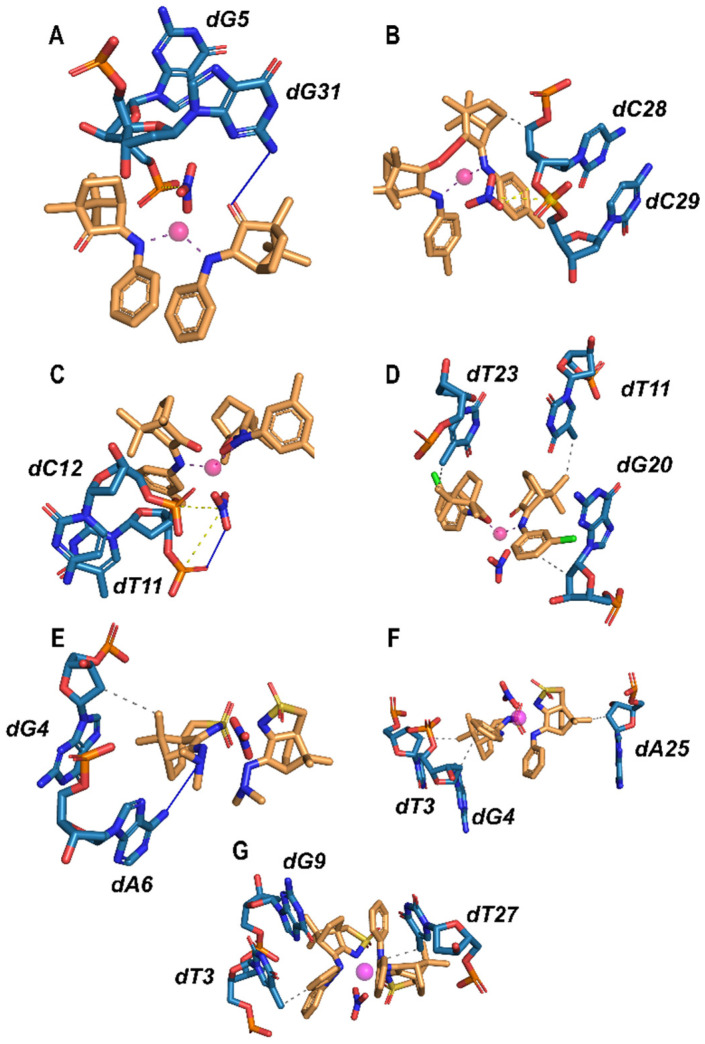
Representation of the major interactions between selected Ag(I) complexes and DNA. Ag(I) complexes **1** (**A**), **4** (**B**), **5** (**C**), **7** (**D**), **12** (**E**), **14** (**F**), and **16** (**G**) are represented as orange carbon sticks, DNA residues as blue carbon sticks, and Ag atoms are shown as magenta spheres. Electrostatic interactions are shown in yellow, H-bonds in blue, and hydrophobic interactions in grey.

The binding interactions include hydrogen bonding, bridging electrostatic interactions, such as interactions with the phosphate backbone or charged residues, and hydrophobic contacts, where nonpolar groups tend to accommodate better near the DNA surface than in solution, minimizing their potential exposure to water. The characteristics of the complex’s co-ligands (NO_3_^−^ or OH^−^) play a highly relevant role in the stabilization of DNA. These interactions, depicted in Figure 8, are discussed hereafter. Docking results indicate that complexes bearing nitrate ligands adopt geometries that favor electrostatic interactions with the DNA phosphate backbone, leading to stronger binding energies. In contrast, replacement of nitrate by hydroxide or a μ-O bridge reduces these interactions, shifting DNA recognition toward weaker π-stacking and hydrogen bonding. This structural trend, NO_3_^−^ > OH^−^ ≫ μ-O^2−^, parallels the electrochemical shifts, highlighting the role of the co-ligand in modulating DNA affinity, even in the absence of direct Ag–DNA coordination.

Complex **1** interacts with DNA through bridging electrostatic interactions between the nitrate (NO_3_^−^) co-ligand and the G5 residue, reinforced by H bonding involving the G31 (r_HA_ = 2.71 Å, r_DA_ = 3.70 Å, θ = 167.3°) residue and the camphor ketone group. The calculated energy value (−252.1 kcal/mol) is consistent with a strong interaction between **1** and DNA. Similarly, the interactions of complexes **4** and **5** with DNA are dominated by bridging between the nitrate and the phosphate groups (C28–C29, **4** and T11–C12, **5**), besides for complex **4** hydrophobic interactions with C28 are also observed. The binding energy calculated for **5** (−274.7 kcal/mol) is among the highest (Table 7) highlighting the relevance of the electrostatic forces in the stabilization of the interactions of the complex with DNA minor groove. Complexes **4** and **5** have in common a nitrate co-ligand responsible for significant electrostatic interactions with DNA, which are absent in the case of complex **23** with a hydroxyl (OH^−^) co-ligand. The binding energy to DNA calculated for **23** (−236.9 kcal /mol) reveals a considerably weaker interaction with the minor groove of DNA than that established by **4** and **5**, showing the relevance of the electrostatic interactions to the increased affinity of DNA. The absence of electrostatic interactions makes complex **23** reliant on weak hydrophobic stabilization.

The binding energy calculated for complex **7** (−276.5 kcal/mol) is similar to that for complex **5** (−274.7 kcal/mol, Table 7) despite the lack of bridging interactions between the phosphate groups of DNA and the nitrate co-ligand. The interaction of **7** with DNA is based on strong hydrophobic contacts between T11, G20 and T23 (major groove), which assure a similar stabilization to that of the electrostatic interactions found for complexes **1**, **4** and **5**. These results show that, at the minor and major grooves, different types of interactions determine the strength of the interaction.

Complexes **12**, **14** and **16**, like complex **7**, fit into the major groove of DNA, although the ligands are of the camphor sulfonimine (**12**, **14**, **16**; Table 3) and camphorimine (**7**; Table 1) types, respectively. All of these (**7**, **12**, **14**, **16**) interact with DNA through relatively strong hydrophobic interactions (**7**, E = −276.5 kcal/mol and **16**, E = −277.6 kcal/mol) that are slightly weaker in the case of **14** (E = −241.2 kcal/mol) and considerably weaker in the case of **12** (E = −224.8 kcal/mol).

The abovementioned complexes have the nitrate co-ligand in common, while distinct camphor ligands are responsible for different electronic and steric properties that drive their accommodation at the DNA major or minor grooves. The nitrate co-ligand (NO_3_^−^) allows optimal electrostatic anchoring via short contacts to the DNA backbone, as exemplified by complexes **1**, **4**, and **5**. These interactions are absent or are weak in OH^−^ and μ-O complexes, explaining their lower docking scores. On the other hand, the camphor carbonyl group aligns the complex for π-stacking and accepts stabilizing H-bonds. In ^B^L-Y ligands, additional sulfonyl oxygen atoms serve as further H-bond acceptors, enhancing minor groove interaction. In what concerns the imine substituents (Y), we find that electron-donating groups (e.g., OH, NH_2_) can donate H-bonds, as verified in complex **23**, while aromatic groups allow additional π-stacking interactions. These peripheral interactions fine-tune the overall binding affinity.

### 3.3. Cytotoxic Activity Versus DNA Binding

The cytotoxic activity is dependent on the cell line and even cells originating from the same tumor can exhibit significant genetic diversity and present different cytotoxic profiles. Therefore, different cell lines have varying sensitivities to cytotoxic agents. This sensitivity is dependent on the different biological features and the presence of mechanisms that protect cells from dying.

It has been formerly reported that silver camphorimine complexes have moderate to high cytotoxic activity, depending on the cell line [19,20]. To complement such data and corroborate the applicability of the Q_[Ag]_/Q_[Ag]+DNA_ relationship, the cytotoxic activity of complexes **19**–**24** was assessed towards the osteosarcoma HOS cell line (Table 8).

The overall results show that the IC_50_ values for cancer cells HOS, A2780, and A2780cisR vary in line with the DNA binding energy calculated by docking and in the opposition to the Q_[Ag]+DNA_/Q_[Ag]_ values (Table 8). Such a pattern shows that complexes with higher reaction coefficient ratios establish stronger interactions with DNA (i.e., binding energies are more negative) and display higher anticancer activity. Therefore, the electrochemical data obtained by cyclic voltammetry, in the presence and absence of CT-DNA, can be used to predict (in an accessible and straightforward way) the relative cytotoxicity of the Ag(I) camphorimine complexes through calculation of the Q_[Ag]+DNA_/Q_[Ag]_ ratio. The same applies to the toxicity calculated through the IC_50_ values for the human dermal fibroblast (HDF), i.e., more negative energies and higher Q_[Ag]+DNA_/Q_[Ag]_ ratios indicate stronger interactions of the complexes with DNA (Table 8). In what concerns the selectivity (S = IC_50(HDF)_/IC_50(HOS)_), the characteristics of the complexes fine-tune the interaction with healthy and cancerous cells. From the complexes of which cytotoxicity was evaluated, complex **14**, which fits into the major groove of DNA with a medium-to-strong energy and displays medium-to-high Q_[Ag]+DNA_/Q_[Ag]_ ratios, reveals the highest selectivity (3.9), in agreement with different affinities, to bind the DNA of healthy (HDF) and cancerous (HOS) cells.

**Figure 9:**
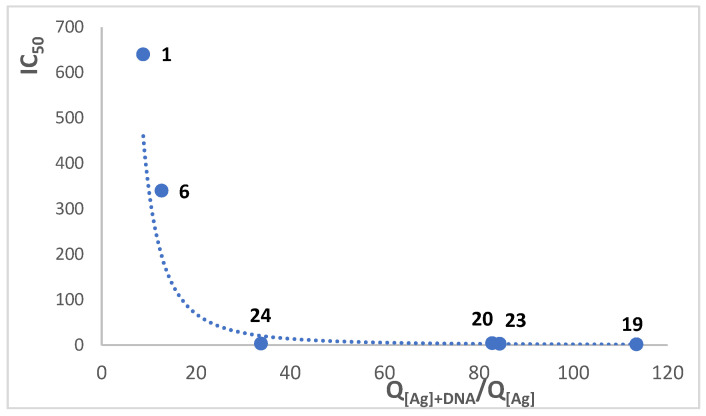
Correlation between the cytotoxic activity (IC_50_) against HOS cancer cells and the reactivity coefficients calculated for Ag(I) camphorimine complexes.

Using the IC_50_ values for HOS cells and the calculated Q_[Ag]+DNA_/Q_[Ag]_ ratio for camphor complexes (Figure 9) an empirical equation was found for Ag(I) complexes (IC_50_ = 70500 × (Q_[Ag]+DNA_/Q_[Ag]_)^−2.314^; *σ*^2^ = 0.9879, not including compounds derived from ^C^L-Z ligands) that allows the prediction of the cytotoxicity (IC_50_) of Ag(I) camphorimine complexes without IC_50_ calculation. In principle, this strategy can be applied to other families of complexes. Within the same HOS dataset more negative docking energies correlate with lower IC_50_ values (Pearson r = –0.65, *p* = 0.11) and higher Q_[Ag]+DNA_/Q_[Ag]_ ratios correlate with lower IC_50_ values (Pearson r = –0.69, *p* = 0.041); both fall in the “strong” correlation range, reinforcing the link between enhanced Ag/DNA interaction and cytotoxic potency.

### 3.4. Morphology and Ag Content of the Products from Interaction with CT-DNA

**Figure 10:**
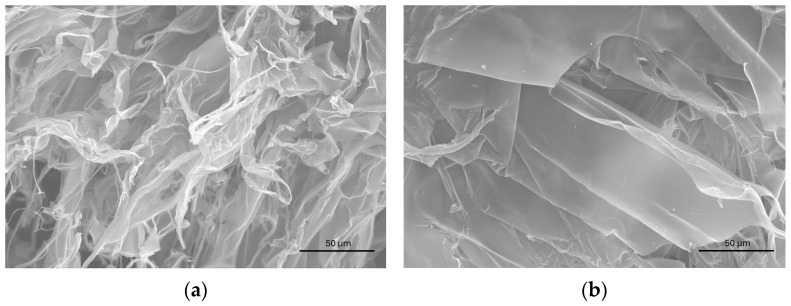
SEM images from CT-DNA. Filament type (**a**) and plate type (**b**).

The material collected from the surface of the Pt electrode during the study of complexes **25** and **27** was analyzed by scanning electron microscopy (SEM) and compared with that of CT-DNA, which exhibited two distinct morphologies: filament (Figure 10a) and plate (Figure 10b).

**Figure 11:**
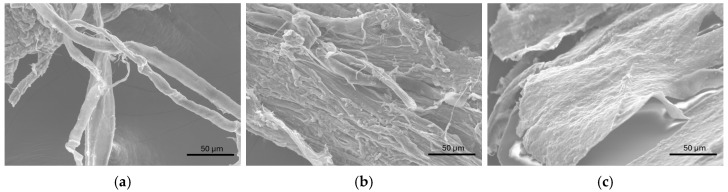
SEM images from the material collected from the surface of a Pt electrode in the study of complexes **25** (**a**,**b**) and **27** (**c**) in the presence of CT-DNA.

The whitish materials adherent to the Pt electrode collected from the study of complexes **25** and **27** in the presence of excess of CT-DNA are filamentous, appearing either as well-separated structures (Figure 11a) or as aggregates (Figure 11b,c).

**Figure 12:**
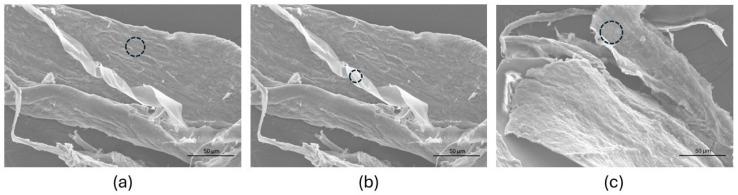
SEM images for **25** (**a**,**b**) and **27** (**c**) showing the region (circles) where EDS analysis was made.

The silver content of the two samples was analyzed by energy dispersive spectroscopy (EDS) (Figure 12). At complex **25**, the highest value (15 at.%) was obtained on the aggregate region (Figure 12a). This value is considerably lower (3 at.%) in the curly region (Figure 12b). At complex **27** (Figure 12c), the highest silver content was 7.4 at.% which is ca. half of that at the aggregate region of complex **25**.

The morphology of the two samples obtained from complexes **25** and **27** are not very different, exhibiting similar morphological features. Despite the differences between the two Ag(I) complexes, this similarity suggests that the interaction of both of these complexes with CT-DNA is similar, leading to an analogous deposition at the surface of the electrode. Such similarity may also imply that experimental conditions, such as excess CT-DNA and the characteristics of the Pt electrode, drive the formation of aggregates with common morphology.

## 4. Conclusions

The cyclic voltammetry studies performed on the silver camphorimine complexes revealed two key cathodic processes—one at higher potentials, corresponding to the Ag(I)→Ag(0) reduction, and another at lower potentials, attributed to the ligand-based reaction. In the presence of CT-DNA, the potentials of the Ag(I)→Ag(0) cathodic waves shift to more negative values, while the potential of the ligand-based waves do not change or shift to more positive potentials. The negative shifts of the potential of the Ag(I)→Ag(0) reduction processes due to the presence of CT-DNA are consistently larger for the nitrate {Ag(NO_3_)} than for the hydroxide {AgOH)} or oxide {Ag_2_(μ-O)} complexes, whereas the positive shift in ligand-based cathodic process is more significant for the hydroxide and oxide than for the nitrate complexes. The reaction coefficient ratio (Q_[Ag+DNA]_/Q_[Ag]_) calculated from the shifts on the potential for the Ag(I)→Ag(0) reduction in the presence and absence of CT-DNA vary by up to two orders of magnitude, with the {Ag(NO_3_)} complexes displaying the highest values. The energy profile and type of interaction established from the complexes with CT-DNA, as determined by molecular docking, show that the more negative energy values, corresponding to stronger interactions with DNA, were found for the complexes with higher Q_[Ag+DNA]_/Q_[Ag]_ ratios, as expected.

The Ag(I) complexes studied exhibit diverse ligand frameworks, donor atoms, and charge distributions, all of which influence their DNA-binding behavior. A key structural difference among these complexes is the nature of the metal co-ligand nitrates (NO_3_^−^) versus hydroxyl (OH^−^) groups. At complexes **4**, **5**, and **7** strong electrostatic interactions between nitrate and DNA phosphates exist which significantly stabilize the complexes, leading to high binding energies (−246 to −276 kcal/mol) no matter the DNA grove to which they fit (**4** and **5**, minor groove; **7** major groove). Complexes of the {Ag(OH)} family, such as **23**, where nitrate was replaced by hydroxyl, lack these electrostatic interactions, leading to weaker binding energy (−236.9 kcal/mol). This trend highlights that electrostatic interactions strongly influence DNA affinity, particularly for minor groove-binding complexes, where close contact with the phosphate backbone facilitates interaction, while binding at the major groove is based on hydrophobic contacts reinforced by hydrogen bonding.

The characteristics and strength of the interactions with DNA correlate well with the cytotoxic activity of the complexes for cell lines A2780, A2780cisR and HOS. A numeric correlation between the reaction coefficients and the cytotoxic activity (IC_50_) against HOS cancer cells supports the effectiveness of cyclic voltammetry to determine whether the anticancer activity of a new synthesized member of a family of complexes is worth evaluation.

## 5. Experimental Section

The ligands were synthesized through condensation of primary amines or hydrazines with camphorquinone (^A^L-Y, ^C^L-Z) or 3-oxo-camphorsulfonylimine (^B^L-Y). The complexes were synthesized under dinitrogen using the procedures described in [21,22,30,31].

The new ligand (3-OHC_6_H_4_)NC_10_H_13_NSO_2_ and the derived complex **17** were synthesized as described below.

Acetonitrile was purchased from Carlo Erba (Milan, Italy) and tetra butyl ammonium tetrafluoroborate from Sigma Aldrich (St. Louis, MO, USA). Borate buffer solution (pH 7.5) was purchased from Fisher Scientific (Waltham, MA, USA) and CT-DNA from Sigma. SEM and EDS-SEM analysis was obtained as an external service from MicroLab—an Electron Microscopy Laboratory at Instituto Superior Técnico—using a Phenom ProX G6 apparatus (Thermo Fisher Scientific, Inc., Waltham, MA, USA).

### 5.1. Synthesis

Ligand (3-OHC_6_H_4_)NC_10_H_13_NSO_2_—3-oxo-camphorsulfonimide (682 mg; 3.0 mmol) was dissolved in ethanol (10 mL) and acidified with CH_3_COOH (0.4 mL). The mixture was stirred for 15 min before 3-aminophenol (329 mg; 3.01 mmol) was added. Upon overnight stirring at 50 °C, a yellow compound formed that was filtered off the solution and dried. Yield 87%. Elem. Anal. (%) for C_16_H_17_N_2_O_3_S Found: C, 60.6; N, 8.8; H, 5.4; S, 10.1. Calc.: C, 60.5; N, 8.7; H, 5.0; S, 10.2. FTIR (KBr, cm^−1^): 3467, 1633, 1594, 1333, 1213. ^1^H NMR (CD_3_CN, δ_ppm_): 7.57 (br, 1H); 7.25 (t, *J* = 8.0 Hz, 1H); 6.69, 6.67 (2d, *J* = 2.3 Hz, 1H); 6.45 (d, *J* = 7.8 Hz, 1H); 6.41 (s, 1H); 3.50, 3.28 (2d, *J* = 13.9 Hz, 2H); 2.95 (d, *J* = 4.8 Hz, 1H); 2.23–2.13 (m, 1H); 1.89–1.71 (m, 3H); 1.03 (s, 3H); 0.87 (s, 3H). ^13^C NMR (CD_3_CN, δ_ppm_): 187.4, 169.0, 158.7, 151.6, 131.3, 113.8, 112.3, 107.9, 64.2, 52.5, 50.6, 47.4, 29.0, 24.6, 20.0, 18.1.

[Ag(NO_3_){(3-OHC_6_H_4_)NC_10_H_13_NSO_2_}_2_] (**17**)—The ligand (3-OHC_6_H_4_)NC_10_H_13_NSO_2_ (318 mg; 0.99 mmol) was dissolved in CH_3_CN (7 mL) and silver nitrate (85 mg; 0.50 mmol) added. The mixture was stirred for 2 h under nitrogen and protected from light. Then, the silver residues were filtered off and the solvent evaporated affording an orange compound. Yield: 82%. Elemental analysis for AgC_32_H_36_N_5_O_9_S_2_·^2^/_5_HNO_3_ Exp.: C, 46.6; N, 9.6; H, 4.4; S, 7.7. Calc.: C, 46.2; N, 9.1; H, 4.3; S, 7.7. FTIR (KBr, cm^−1^): 3462, 1667, 1636, 1384, 1345, 1162. ^1^H NMR (400 MHz, CD_3_CN, δ ppm): 7.48 (br, 1H); 7.25 (t, *J* = 8.0 Hz, 1H); 6.70, 6.68 (2d, *J* = 1.9 Hz, 1H); 6.46 (d, *J* = 7.8 Hz, 1H); 6.42 (s, 1H); 3.51, 3.28 (2d, *J* = 14 Hz, 2H); 2.95 (d, *J* = 4.7 Hz, 1H); 2.34–2.24 (m, 1H); 1.89–1.72 (m, 3H); 1.05 (s, 3H); 0.88 (s, 3H). ^13^C NMR (400 MHz, CD_3_CN, δ ppm): 187.6, 169.3, 158.9, 151.8, 131.2, 113.8, 112.4, 107.9, 64.2, 52.5, 50.6, 47.4, 29.0, 24.5, 20.0, 18.1.

### 5.2. Cyclic Voltammetry Experiments

A three-compartment cell equipped with Pt wire working and secondary electrodes interfaced with a VoltaLab PST050 equipment (Radiometer Analytical S.A. made in Neuilly-Plaisance, France) was used. The cyclic voltammograms were obtained at 200 mV/s from the CH_3_CN/NH_4_BF_4_ solutions (0.10 M) that were used as electrolytes. The electrode double-layer was renewed through nitrogen bubbling. The window of potential was established using a solution of the freshly prepared electrolyte. No waves were observed for DNA under the employed experimental conditions.

### 5.3. Cytotoxic Assays

The evaluation of the cytotoxic activity of complexes **19**–**24** towards the human osteosarcoma cells HOS (ATCC, CRL-1543), which are a mixture of fibroblast and epithelial-like cells isolated from the bone of a white female patient with osteosarcoma, was made with cells cultured in DMEM + GlutaMAX medium supplemented with 10% FBS at 37 °C and 5% CO_2_ in a humidified atmosphere. For the assays, cells were seeded in 96-well plates at a density of 2 × 10^4^ cells/ 200 μL medium and allowed to adhere 24 h. The complexes were dissolved in DMSO to prepare a stock solution of 10 mM and then in culture medium to prepare serial dilutions in the range of 0.1–100 µM. After 24 h, the cellular viability was measured using the MTT assay, as previously described [19]. The percentage of cellular viability was assessed measuring the absorbance at 570 nm using a Varioskan LUX (Thermo Fisher Scientific, Waltham, MA, USA) scanning multimode reader. The IC_50_ values were calculated from dose–response curves using the GraphPad Prism 8.0 software.

### 5.4. Docking Calculations

The Ag complexes were first geometry-optimized using the MMFF94 force field [32,33]. A fully blind, rigid-body docking approach was employed in Hex, utilizing fast Fourier transform (FFT)-based correlation algorithms to evaluate shape complementarity and electrostatic interactions between the molecules. To explore large biomolecule docking, Hex’s macro docking approach was applied, defining the search space with 13 docking spheres, each with a 15 Å radius, and a maximum docking distance of 25 Å, spanning the entire DNA molecule. The shape and electrostatic correlation scoring functions were used to assess non-covalent interactions, electrostatics, and steric complementarity. The ligand and receptor rotational search ranges were set to 45°, and the 5D FFT mode was enabled to enhance the search resolution, generating 5000 docking solutions. Docking solutions were ranked based on their interaction energy scores, considering shape complementarity and electrostatic interactions. The best docking conformations were analyzed to determine binding modes, with a particular focus on non-covalent interactions and groove binding. Notably, no docking solutions involving intercalation were observed among the top 50 docked poses, suggesting that the Ag complexes predominantly interact with the major and minor grooves of the DNA rather than intercalating between base pairs. DNA–ligand interactions were analyzed with PLIP [34], PyMOL [35], and in-house scripting based on MDAnalysis [36,37].

## Figures and Tables

**Table 8:** Cytotoxicity versus reaction coefficients for the interaction of complexes with DNA.

		IC_50_ (µM)				Q_[Ag]+DNA_/Q_[Ag]_	E ^d^ kcal/mol
Complex	HOS	SI ^a^	A2780	A2780cisR ^c^	HDF		
**1**	8.8 ± 2.5^b^	2.1	3.53 ± 0.90 ^b^		18.1 ± 4.8	6.4 × 10^2^	−252.1
**6**	12.7 ± 4.5^b^	1.4			17.4 ± 3.6	3.4 × 10^2^	−280.7
**11**	3.1 ± 0.9^a^	2.7			8.5 ± 2.7	1.1 × 10^2^	−252.9
**12**			1.11 ± 0.26 ^c^	1.17 ± 0.22		1.0 × 10^1^	−224.8
**14**	8.29 ± 2.0^b^	3.9	0.76 ± 0.29 ^c^	0.51 ± 0.10	32.3 ± 8.7	1.1 × 10^2^	−241.2
**16**			0.65 ± 0.17 ^c^	0.67 ± 0.19		1.0 × 10^1^	−277.6
**19**	113.4 ± 16.3		10.4 ± 2.9			1.7	−219.8
**20**	84.4 ± 25.6	0.9			76.9 ± 14.3	3.0	
**21**	6.7 ± 2.8	1.6			10.6 ± 3.5	3.2	238.6
**23**	82.8 ± 15.1	1.0			82.4 ± 13.5	4.1	−236.9
**24**	33.8 ± 13.4	0.9	0.66 ± 0.28 ^b^		30.6 ± 8.5	3.2	

^a^ IC_50_(HDF)/IC_50_(HOS). ^b^ Ref. [19]. ^c^ Ref. [29]. ^d^ Binding energies as computed by docking.

## Data Availability

The original contributions presented in this study are included in the article. Further inquiries can be directed to the corresponding author.
